# Egalitarian reward contingency in competitive games and primate prefrontal neuronal activity

**DOI:** 10.3389/fnins.2015.00165

**Published:** 2015-05-15

**Authors:** Takayuki Hosokawa, Masataka Watanabe

**Affiliations:** ^1^Department of Physiological Psychology, Tokyo Metropolitan Institute of Medical ScienceTokyo, Japan; ^2^Division of Systems Neuroscience, Tohoku University Graduate School of Life SciencesSendai, Japan

**Keywords:** egalitarian reward-contingency, prefrontal cortex, monkey, neuron, competition, game

## Abstract

How people work to obtain a reward depends on the context of the reward delivery, such as the presence/absence of competition and the contingency of reward delivery. Since resources are limited, winning a competition is critically important for organisms' obtaining a reward. People usually expect ordinary performance-reward contingency, with better performers obtaining better rewards. Unordinary reward contingency, such as egalitarianism (equal rewards/no-rewards to both good and poor performers), dampens people's motivation. We previously reported that monkeys were more motivated, and neurons in the lateral prefrontal cortex (LPFC) showed higher outcome-related activity in a competitive than in a noncompetitive game (Hosokawa and Watanabe, 2012). However, monkey's behavior and LPFC neuronal activity have not been examined in a competitive situation with an unordinary performance-reward contingency. Also, the fixed performance-reward contingency in the previous study did not allow us to examine effects of win/loss separately from those of reward/no-reward on prefrontal neuronal activity. Here, we employed the egalitarian competitive situation in which both the winner and loser, or neither of them, got a reward as well as the normal competitive situation in which only the winner got a reward. Monkey's behavioral performance greatly deteriorated in trials with the egalitarian outcome conditions. LPFC neurons showed activities that reflected the normal or egalitarian outcome condition while very few neurons coded win/loss independent of reward/no-reward. Importantly, we found neurons that showed reward-related activity in the normal, but not in the egalitarian outcome conditions, even though the same reward was given to the animal. These results indicate that LPFC may play an important role in monitoring the current reward contingency and integrating it with the performance outcome (win-loss) for better performing the competitive game, and thus for better survival.

## Introduction

How people work to obtain a reward depends not only on its quality and quantity, but also on the context of its delivery, such as the presence or absence of competition and the contingency of reward delivery. Since resources are limited in the natural environment, competition is inevitable, and competing successfully is critically important for animal's obtaining a reward. In everyday life, people usually expect ordinary performance-reward contingency, with better performers receiving better rewards (e.g., money, fame, and acknowledgment). Unordinary reward contingency, such as egalitarianism (equal rewards/no-rewards to both good and poor performers), dampens people's motivation (Friedman and Friedman, 1990; Fehr and Schmidt, 1999). We previously reported that monkeys were more motivated to play a game to obtain a reward in the competitive situation, where two monkeys competed against each other in a shooting game, than in the noncompetitive situation, where one monkey played the game without a rival, and that a group of LPFC neurons showed higher outcome-related activity in the competitive situation (Hosokawa and Watanabe, 2012). Reward-related neuronal activities of the prefrontal cortex are reported to reflect the social context regarding whether an individual's task performance is associated with only the individual's own reward or with both the individual's and a task-uninvolved partner's rewards (Azzi et al., 2012; Chang et al., 2012). However, it has not been clear how unordinary reward contingency, such as egalitarian reward contingency, affects monkey's behavior and LPFC neuronal activity in competitive situations.

In the previous study the performance-reward contingency was fixed: winning and losing a competition always led to the presence and absence of a reward, respectively. Thus, we were unable to examine effects of the performance outcome (win-loss) separately from those of the presence/absence of the reward on prefrontal neuronal activity. Prefrontal neurons are known to code the correctness of one's own response (Watanabe, 1989) independent of the presence/absence of the reward. Also, a group of prefrontal neurons selectively encode others' action (Yoshida et al., 2011). So, we were also interested in examining whether prefrontal neurons are concerned with coding reward-independent performance outcome; whether they code the winning or losing of the game independent of the presence/absence of the reward.

To address these questions, we used a competitive video game with egalitarian outcome conditions, in which both the winner and loser or neither of them received a reward, besides the game with a normal outcome condition, in which only the winner received a reward. In the egalitarian outcome conditions, winning and losing experiences were independent of the presence and absence of a reward. We predicted that the egalitarian reward contingency in competition would greatly affect the monkey's behavior and hypothesized that LPFC neurons would play important roles in distinguishing the context of reward delivery between the normal and egalitarian reward contingencies in competition. We also predicted that there would be LPFC neurons that code the win/loss of the game independent of the presence/absence of the reward.

## Materials and methods

### Animal subjects

We used three Japanese macaques (*Macaca fuscata*): monkey H, 7.1 kg; monkey S, 6.4 kg; and monkey P, 8.2 kg). The same monkeys were used in the previous study (Hosokawa and Watanabe, 2012). All experiments were conducted in accordance with the National Academies Press (USA) guidelines for animal experiments and were approved by the ethics committee of our institute. During the experiments, which were conducted on weekdays, the monkeys obtained all of their fluid by playing games, whereas they were given free access to water during the weekend.

### Competitive shooting game with normal and egalitarian outcome conditions

**Figure 1:**
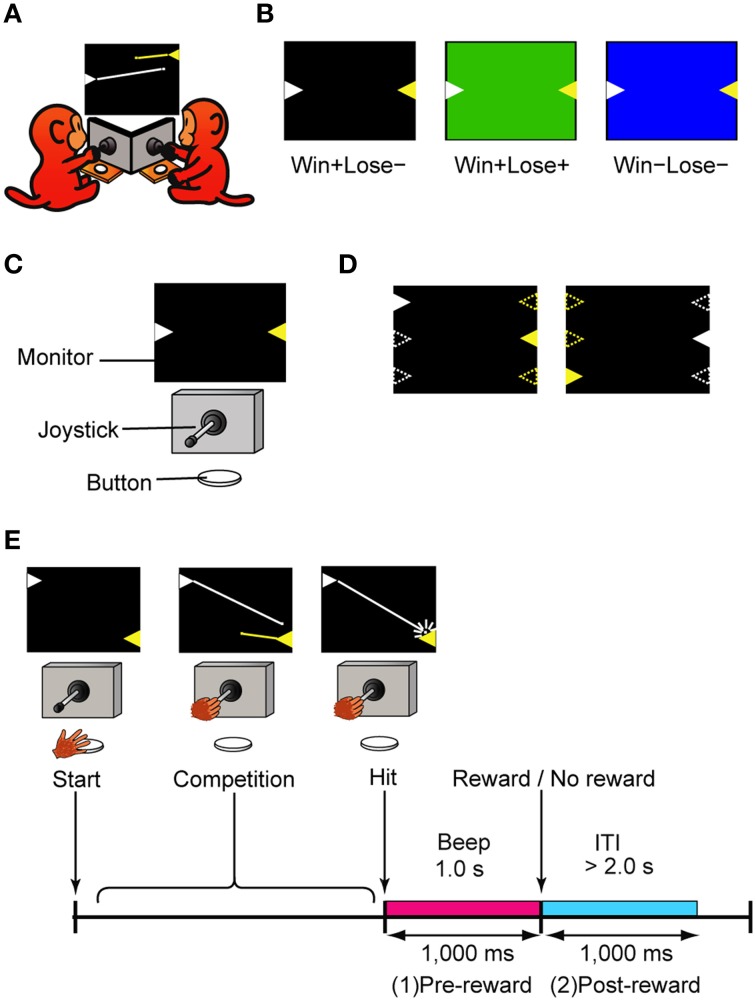
**The competitive video game. (A)** Schematic diagram of the game. Each monkey shot bullets from the turret (triangle) of its color, which was fixed for each monkey. The lines from the turrets represent the trajectories of the bullets and did not appear in the actual game. **(B**) Three types of trials used in this study: Win+Lose**−**, Win+Lose+, and Win**−**Lose−trials. In Win+Lose**−** trials (black background), the winner got a reward and the loser did not. In Win+Lose+ trials (green background), both the winner and the loser got a reward. In Win**−**Lose**−** trials (blue background), neither the winner nor the loser got a reward. **(C)** Experimental setup. There was a joystick and a button in front of each monkey. Monkeys shared one PC monitor. **(D**) Position and spatial configuration of the turrets. The turret positions were randomly selected from top, middle, or bottom, and left or right. These positions changed from trial to trial, but they were fixed within a trial. Open triangles represent possible positions at which turrets could appear. **(E**) Time course of the competitive shooting game and the analysis periods. ITI, Intertrial interval.

Two monkeys faced a computer monitor (LCD-AD193VB, I-O Data, Ishikawa, Japan), arranged at an angle so that they could see each other (Figure 1A). In front of each were a horizontally protruding joystick (40JBK-YO-20R2G, Sakae Tsushin Kogyo Co., LTD., Kanagawa, Japan) and a button (OBSA-60UM, Sanwa Denshi Co., LTD., Osaka, Japan) (Figure 1C). When both of the monkeys pushed their own button, a trial started and two colored triangles (white and yellow) appeared on the left and right sides of the monitor, facing each other (Figure 1D). Each of the triangles represented a turret from which a bullet could be launched in the direction that the joystick was tilted. A bullet was launched when the tilt angle of the joystick exceeded a threshold (22.5 degrees from the neutral position). The trajectory of each bullet was linear and could not be changed after the bullet was launched. Because the skill of playing the games was different among monkeys, the speed of the bullet was tailored for each monkey in the range between 25 and 33 degrees/s to keep the winning rate about 50%. We trained the monkeys to tilt the joystick and shoot at the turret (target) on the other side. Once the monkey shot a bullet, another bullet could not be launched until the first bullet went out of view. The color of the turret was fixed for each monkey: white for monkeys H and S, and yellow for monkey P. The positions of the turrets were randomly selected from top, middle, or bottom, and left or right (Figure 1D). These positions changed from trial to trial but were fixed within a trial. When a monkey hit the target, a 1-s beep was presented and followed or not followed by a reward. The beep was always the same irrespective of which monkey won the game. During the beep, the winner's turret flashed on and off, and the loser's turret vanished gradually (it looked like it was collapsing). The monkeys had to push the button to advance to the next trial after a trial of competition finished (after a bullet hit the target). To ensure that both monkeys were motivated to play the game, the next trial did not start until both monkeys had pushed the button. The inter-trial interval (ITI) differed depending on the monkey's button-pushing response time but was set to be greater than 2 s.

The monkey that made the first successful shot (hitting the target) was the winner, but whether or not the monkeys would obtain a reward was determined not only by which monkey won the competition but also by the trial condition indicated by the background color of the monitor (Figure 1B). When it was black, the winner got a reward (0.3 ml of grape juice) and the loser did not get any reward (W+L− trials, *normal competitive reward condition*). When it was green, both the winner and the loser got a reward (W+L+ trials, *egalitarian reward condition*). When it was blue, neither the winner nor the loser got any reward (W−L− trials, *egalitarian no-reward condition*). In W−L− trials, to advance to the next trial, the current trial had to be terminated by either monkey's winning response. These three types of trials were intermingled and randomly presented in the same session. When neither monkey won the competition within 25 s, that trial was terminated and the condition of the next trial was the same. During the training phase, we also tried to get the monkeys to play the game in a purple-background condition where the winner got no reward and the loser got a reward (W-L+ trials, where the monkeys had to lose the game to obtain a reward). We were, however, not successful – the monkeys refused to play the game.

The monkeys also played the normal competitive and noncompetitive games that were used in the previous study (Hosokawa and Watanabe, 2012). The normal competitive game was almost the same as W+L− trials except that a white or yellow cross, instead of a flashing turret and gradually vanishing one, was presented on the monitor during the beep. In the noncompetitive game, the monkey played the game without a rival. The monkeys played these games in separate sessions.

### Surgery

After the training was completed, surgery was conducted in sterile conditions. The monkey was surgically prepared under sodium pentobarbital anesthesia (20 mg/kg body weight, i.v.). A stainless steel recording chamber (20 × 20 mm square) was implanted as a microdrive receptacle on the skull stereotaxically over the prefrontal cortex, and a head-restraining device (15 mm in diameter) was attached to the skull with dental acrylic. The monkey was given antibiotics every day for a week after the surgery.

### Recordings

For technical reasons, we recorded neuronal activity in the monkey positioned to the left of the other one. Single-neuron activity in the LPFC was recorded extracellularly using tungsten electrodes (2.0–8.0 MΩ, FHC, Bowdoinham, ME). An electrode was advanced with a hydraulic microdrive (MO-95C, Narishige, Tokyo, Japan) through a stainless steel guide tube. Neuronal activities were converted into pulses using a spike waveform detector (Multispike Detector, Alpha Omega Engineering, Nazareth, Israel). We recorded activity in the right hemisphere of monkey S and in both hemispheres of monkey H while they were playing the games. Monkey P was always the competitor for monkeys H and S, which never competed against each other because both of them were trained with the same turret color (white). The recording area covered both the dorsal and ventral banks of the principal sulcus (**Figure 8A**), and was determined in reference to magnetic resonance images (whole-brain coverage, slice thickness 2 mm, Siemens, Sonata 1.5T).

We monitored the eye position of monkeys H and S with an infrared eye-camera system (sampling rate, 4 ms; R-22C-1, ISEYO Electronic, Tokyo, Japan), but did not restrict or control their eye movements.

### Statistical analysis

We used the first-shot hit rate (the ratio of the number of trials in which the first shot was successful to the total number of trials) as a measure of the accuracy of hitting, used the latency from the start of each trial to the time of the first bullet shot as a measure of the quickness of shooting, and compared them among the trial types (Bonferroni-corrected two-tailed *t*-test, *p* < 0.05). In the calculation of the first-shot hit rate, “loss” trials in which the bullet was launched in the right direction and would have hit the target if it had been launched earlier were considered successful.

We analyzed neuronal data in relation to winning/losing a competition, as well as the presence/absence of a reward. We examined the neuron activity in two periods: that during the 1000 ms before the reward delivery (pre-reward period), and that during the 1000 ms after the reward delivery (post-reward period, Figure 1E).

We used stepwise multi-linear regression with 10 possible explanatory variables: (1) Reward factor (presence of reward: 1, absence of reward: −1), (2) Win/Lose factor (won: 1, lost: −1), (3) the interaction between the reward and win-lose factors (Reward × Win/Lose factor), (4) background color factor for black (Black factor, black trials: 1 other trials: 0), (5) background color factor for green (Green factor, green trials: 1 other trials: 0), (6) background color factor for blue (Blue factor, blue trials: 1 other trials: 0), (7) the interaction between the Win/Lose and Black factors (Win/Lose × Black), (8) the interaction between the Win/Lose and Green factors (Win/Lose × Green), (9) the interaction between the Win/Lose and Blue factors (Win/Lose × Blue), and (10) the latency of the first shot (First shot factor). If a neuron fitted a regression model in both analysis periods, we used the result in the period in which the Akaike Information Criterion (AIC) was smaller.

For the neurons that were recorded in both the competitive and noncompetitive games that were used in the previous study (Hosokawa and Watanabe, 2012), we analyzed the neuronal data by two-way ANCOVA (competition and reward factors). Details are provided elsewhere (Hosokawa and Watanabe, 2012).

## Results

### Monkey behavior in trials with normal and egalitarian outcome conditions

**Figure 2:**
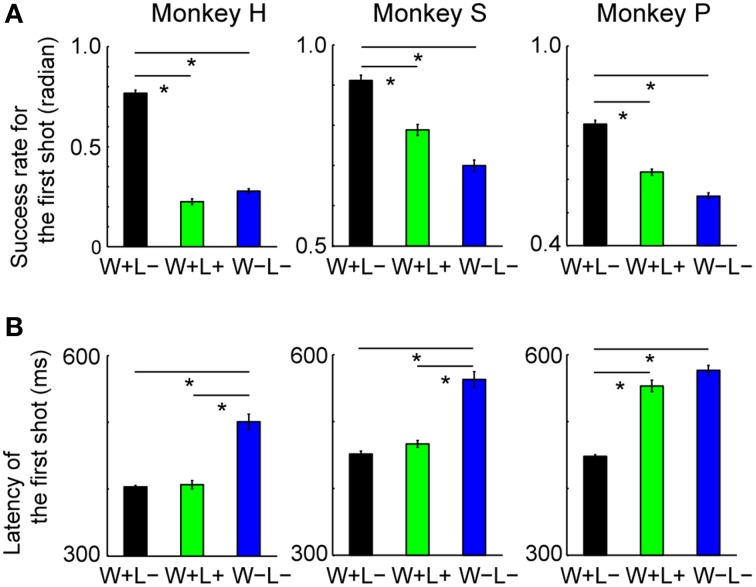
**Behavioral data in the competitive game. (A)** Hit rate for the first shot (mean ± SEM). Data were normalized by the arcsine transformation before statistical analyses. One-Way ANOVA demonstrated significant differences in the first-shot hit rate among the three types of trials [monkey H, *F*_(2, 294)_ = 488.2, *p* < 10^−93^; monkey S, *F*_(2, 471)_ = 61.7, *p* < 10^−23^; monkey P, *F*_(2, 768)_ = 120.8, *p* < 10^−45^]. Post hoc paired comparisons were conducted using a Bonferroni-corrected two-tailed *t*-test (^*^p < 0.05). Monkey H, *n* = 99; Monkey S, *n* = 158; Monkey P, *n* = 257. **(B)** Latency of the first shot (mean ± SEM). Means of the median in each session were compared among the trial types. One-Way ANOVA demonstrated significant differences in latency among the three types of trials [monkey H, *F*_(2, 294)_ = 53.4, *p* < 10^−19^; monkey S, *F*_(2, 471)_ = 60.2, *p* < 10^−23^; monkey P, *F*_(2, 768)_ = 105.9, *p* < 10^−40^]. *Post-hoc* paired comparisons were conducted using a Bonferroni-corrected two-tailed *t*-test (^*^p < 0.05). Monkey H, *n* = 99; Monkey S, *n* = 158; Monkey P, *n* = 257.

We found significant behavioral differences in accuracy and quickness of the first shot between the trial types: the monkeys performed more accurately and quickly in Win+Lose**−** (W+L−) trials than in Win+Lose+ (W+L+) and Win**−**Lose**−** (W−L−) trials. The hit rate for the first shot (the ratio of the number of trials in which the first shot was successful to the total number of trials) was significantly higher in W+L− trials than in the other trials (Figure 2A; Bonferroni-corrected, two-tailed *t*-test, *p* < 0.05). We did not find a significant difference in the hit rates between W+L+ and W−L− trials in either monkey (Figure 2A; Bonferroni-corrected, two-tailed *t*-test, *p* > 0.05). The latency of the first shot was shortest in W+L− and longest in W−L− trials in all monkeys (Figure 2B, Bonferroni-corrected *t*-test, *P* < 0.05), although there was no significant difference between W+L− and W+L+ trials in two monkeys, or between W+L+ and W−L− trials in one monkey. These findings suggest that the monkey's motivation was higher in trials with the normal competitive reward condition (i.e., when they had to win to obtain a reward) than in trials with the egalitarian outcome conditions (i.e., when the presence/absence of reward was independent of winning or losing).

**Figure 3:**
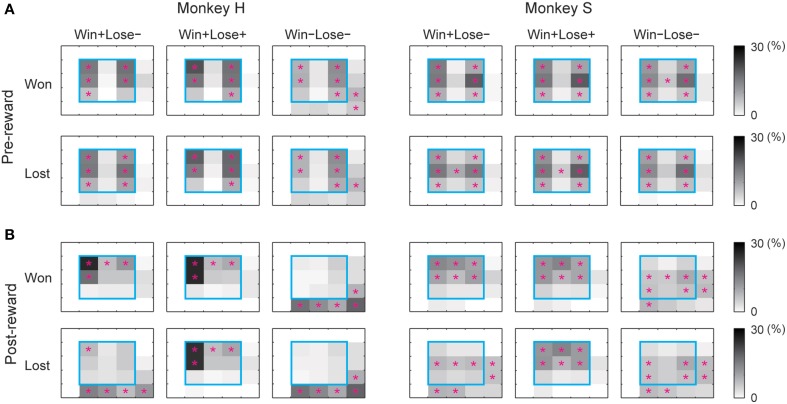
**Percentage of time that the monkey gazed at each section inside and outside the monitor during the analysis periods.** (**A**) pre-reward, (**B**) post-reward periods. For each analysis period, we calculated the percentage of total time that the monkey looked at each section inside the monitor (divided into 3 × 3 sections) and outside the monitor (resulting in 5 × 5 sections in total). The percentage of total gaze time is shown in grayscale separately for won and lost trials. The cyan square represents the area of the monitor. A magenta asterisk indicates that the gaze duration in that section was significantly longer than that calculated from randomized data (bootstrap method, *p* < 0.05, 1000 randomizations).

We also examined eye positions in the two monkeys during the game. Figure 3 shows the percentage of time that each monkey looked at each section inside and outside the monitor. In the pre-reward period, the monkeys looked significantly more at the left and right parts of the monitor, probably because they looked at their own or their opponent's turret to check which of them had won the competition. In the post-reward period, they looked significantly more at the upper part of the monitor when they received a reward (i.e., won in W+L− trials and won or lost in W+L+ trials) and in the right and lower directions outside the monitor when they did not receive a reward (i.e., lost in W+L− trials and won or lost in W−L− trials). During the inter-trial period, there was no tendency for the monkeys to look at specific parts of the monitor. When the monkey did not obtain the reward, it appeared to look at the opponent to check whether the opponent had received a reward, since the opponent was located in the direction of the right lower corner of the monitor. These results indicate that each monkey paid attention to the other monkey when it did not receive a reward.

### Neuronal responses explained by stepwise regression

We obtained a sufficient number of trials for statistical analyses of the activity of 257 neurons (99 in monkey H and 158 in monkey S), and we analyzed their responses during pre- and post-reward periods by stepwise linear regression analysis with 10 explanatory factors (see Materials and Methods and Table 1). The responses of most neurons (238/257, 92.6%) were explained by these 10 factors. We subclassified each type of neuron into positive and negative types depending on whether the beta value of the corresponding factor was positive or negative (Table 1).

**Table 1:** **Number of neurons selected for each factor in the stepwise regression**.

	**Type**	**Subtype**	**Pre-reward period**	**Post-reward period**	**Either period**	**Total number**
1	Reward	Positive	22	14	36	91
		Negative	21	34	55	
2	Win/Lose	Positive	5	3	8	21
		Negative	8	5	13	
3	Reward x	Positive	8	3	11	18
	Win/Lose	Negative	4	3	7	
4	Black	Positive	16	6	22	31
		Negative	6	3	9	
5	Green	Positive	8	9	17	33
		Negative	10	6	16	
6	Blue	Positive	11	13	24	68
		Negative	36	8	44	
7	Win/Lose x	Positive	18	3	21	48
	Black	Negative	14	13	27	
8	Win/Lose x	Positive	9	2	11	20
	Green	Negative	6	3	9	
9	Win/Lose x	Positive	6	5	11	19
	Blue	Negative	4	4	8	
10	First shot	Positive	9	10	19	28
	latency	Negative	3	6	9	

Note that some neurons were classified into two or more types.

**Figure 4:**
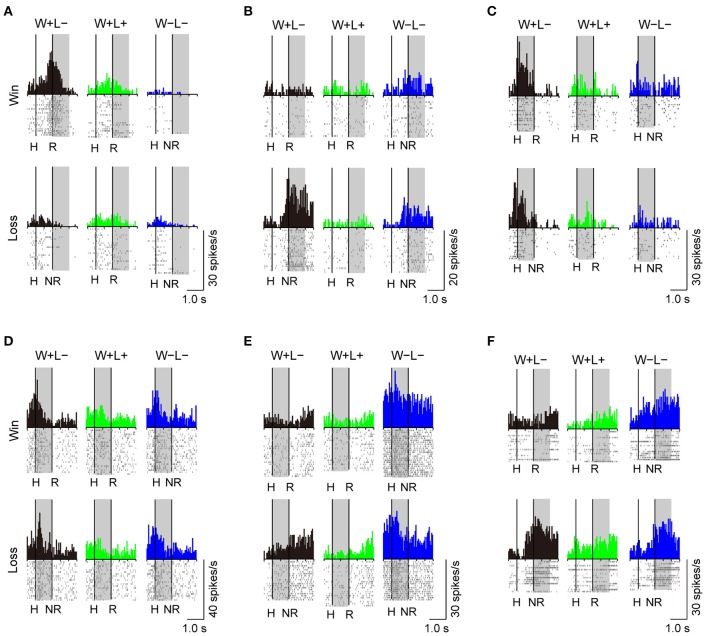
**Examples of LPFC neurons that showed differential activity depending on the trial type.** (**A**) positive Win/Lose x Black, (**B**) negative Win/Lose x Black, (**C**) positive Black, (**D**) negative Green, (**E**) positive Blue, and (**F**) negative Reward type neurons (“positive” and “negative” indicate neurons with positive and negative beta values, respectively. See Materials and Methods). The left vertical line in each display indicates the time of a hit by either monkey. The right vertical line indicates either the time of a reward delivery (reward trials) or 1 s after a hit (no-reward trials). Each shaded area indicates the period when the typical activity of each type was observed. H, Hit; R, reward delivery; NR, 1 s after a hit.

**Figure 5:**
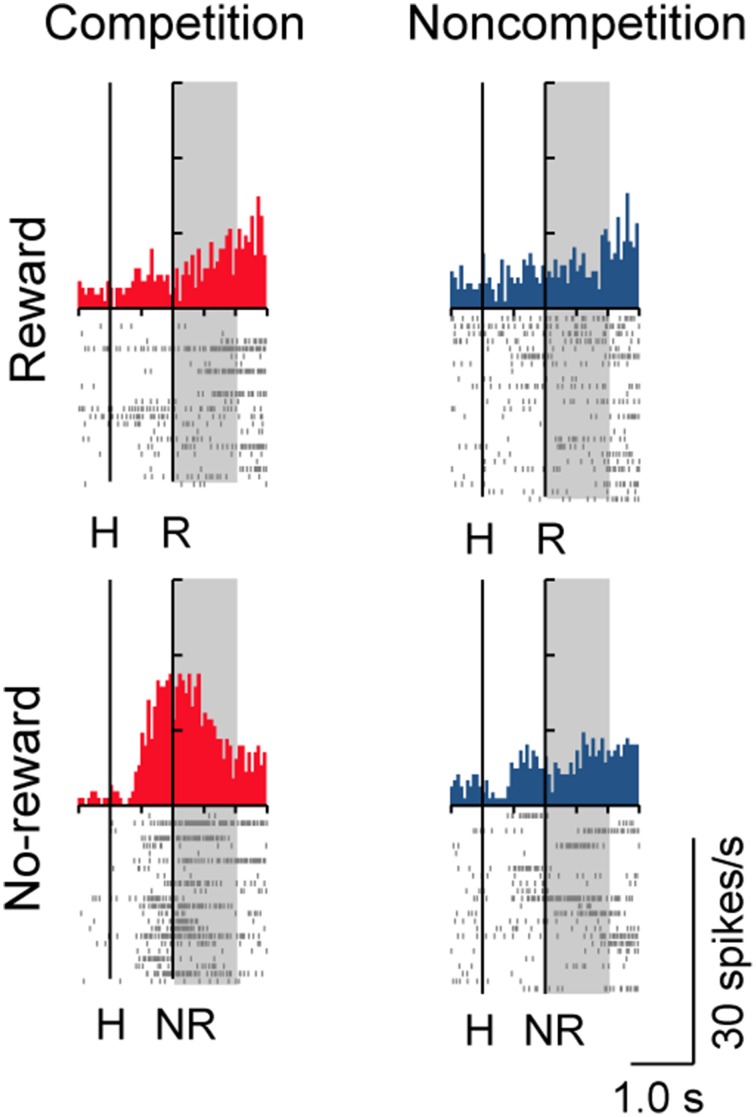
**Activity of a Reward type neuron in competitive and noncompetitive games.** This is the same neuron as in Figure 4F. This neuron showed higher no-reward-related activity in the competitive than in the noncompetitive game.

A substantial number of LPFC neurons showed significantly higher activity in the presence or absence of reward only in trials with the normal competitive reward condition (Win/Lose × Black type, *n* = 48). The neuron in Figure 4A (positive Win/Lose × Black type) showed a reward-related response in the post-reward period. The reward-related response in W+L− trials was much higher than that in W+L+ trials, even though the monkey received the same reward in these trials, suggesting that the response of this neuron did not simply reflect a reward delivery. The response of the neuron in Figure 4B (negative Win/Lose × Black type) was much higher in W+L− trials than that in W−L− trials when there was no reward. Many LPFC neurons reflected the normal/egalitarian reward contingency indicated by the background color irrespective of whether the monkey won or lost a competition: Black (W+L−, *n* = 31), Green (W+L+, *n* = 33), and Blue (W−L−, *n* = 68) types. The neuron in Figure 4C (positive Black type) showed higher activity during the pre-reward period in W+L− trials than in W+L+ and W−L− trials. The neuron in Figure 4D (negative Green type) showed lower activity in W+L+ trials than in W+L− and W−L− trials. The neuron in Figure 4E (positive Blue type) showed higher activity in W−L− trials than in W+L− and W+L+ trials. Also many LPFC neurons showed activity depending on the presence or absence of the reward: Reward type neurons (*n* = 91). The neuron in Figure 4F (negative Reward type) showed higher activity in no-reward trials during the post-reward period. However, the same neuron showed differential no-reward activity between the competitive and noncompetitive games, which were the game conditions used in the previous study (Hosokawa and Watanabe, 2012) (Figure 5). Among 78 Reward type neurons that we were able to record in both the competitive and noncompetitive conditions, 42 (53.8%) showed differential outcome-related activity between the competitive and noncompetitive conditions (the main effect of competition in Two-Way ANCOVA, *p* < 0.05. see Materials and Methods), suggesting that the activity of these neurons did not simply reflect the presence or absence of the reward.

**Figure 6:**
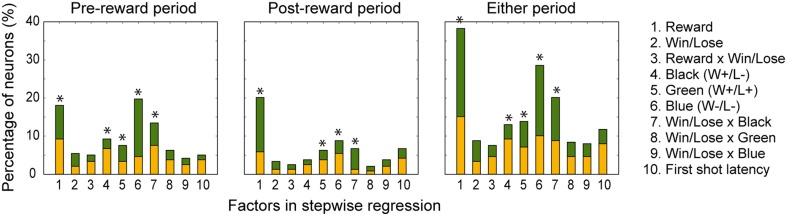
**Percentage of neurons selected for each factor in the stepwise regression in the pre-reward and post-reward periods and either period.** The yellow part of the bars indicates the percentage of neurons with a positive beta value, and the green part indicates the percentage of those with a negative beta value. 1, Reward; 2, Win/Lose; 3, Reward x Win/Lose; 4, Black; 5, Green; 6, Blue; 7, Win/Lose x Black; 8, Win/Lose x Green; 9, Win/Lose x Blue; and 10, First-shot-latency factors. An asterisk indicates that the percentage of neurons for the factor was significantly higher than that calculated from randomized data (binomial test, *p* < 0.05; 1000 randomizations).

**Figure 7:**
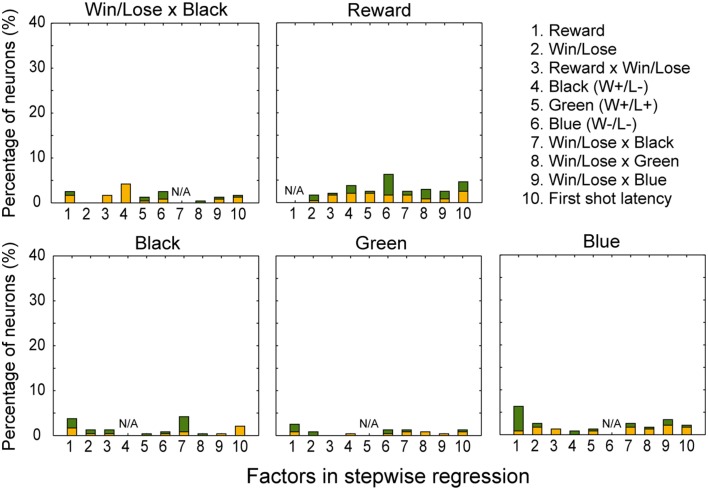
**Percentage of neurons coding each factor concurrently with Win/Lose x Black, Black, Green, Blue, and Reward factors, respectively, in the stepwise regression.** For example, about 4% of neurons were selected for both Reward and Black factors (1 in the panel for Black). Conventions are the same as in Figure 6.

**Figure 8:**
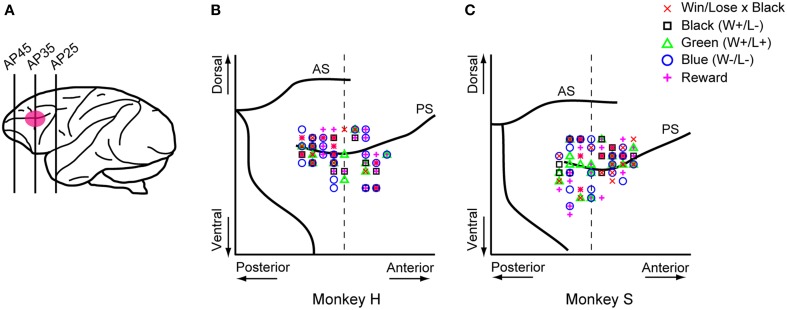
**Sites where each type of neuron was recorded. (A)** Recording areas. Recording areas are highlighted with a pink ellipse on a lateral view of the monkey brain. We recorded LPFC neurons in both the upper and lower banks of the principal sulcus. Most of the recordings were made in the region between AP30 and AP40. AP, anterior-posterior. **(B,C**) Recording sites of each type of neuron were mapped onto comparative locations of the right hemisphere of each monkey's brain based on magnetic resonance images. Red crosses, black squares, green triangles, blue circles, and magenta plus signs represent Win/Lose x Black, Black, Green, Blue, and Reward type neurons, respectively. There were no biases in the recorded areas for each type of neuron across four parts separated by the dorsal and ventral parts from the principal sulcus and the anterior and posterior parts from AP 35 (chi-squared test, *p* > 0.05). The vertical dashed line represents AP 35. AS, arcuate sulcus; PS, principal sulcus.

In both the pre- and post-reward periods the percentage of neurons coding either of the Win/Lose × Black, Green, Blue, or Reward factors (both positive and negative subtypes) was significantly higher than that calculated from randomized data (Figure 6, binomial test, *p* < 0.05; 1000 randomizations). Likewise, the percentage of neurons coding the Black factor (only positive subtype) was significantly higher than that calculated from randomized data in the pre-reward period (Figure 6, binomial test, *p* < 0.05; 1000 randomizations). In other words, there were more neurons of these types than would be expected by chance. Neuronal responses of Win/Lose × Black, Black, and Blue types were more frequent during the pre- than post-reward period (chi-squared test, *p* < 0.05, Figure 6 and Table 1). This may be because the information about whether the reward contingency was normal or egalitarian was more important for the monkeys before than after the reward delivery. Some neurons were classified into two or more types, but we did not find any tendency for neurons to concurrently encode two (or more) specific factors (chi-squared test, *p* > 0.05; Figure 7 and Table 1). There was no clear segregation of any type of neurons in the LPFC (Figures 8B,C).

## Discussion

This study showed that a monkey's behavior and prefrontal neuronal activity depend on whether the reward contingency is normal or egalitarian. The monkey's behavioral performance deteriorated in trials with the egalitarian outcome conditions. In W−L− trials, where winning the game had nothing to do with obtaining a reward, it is reasonable that the monkeys lost their motivation: they were not eager to win the game and might even have wished for the opponent to win and terminate the trial. Interestingly, their motivation was also reduced in W+L+ trials even though they could obtain a reward by winning a competition. The situation in W+L+ trials, where there was no need for the monkeys to compete to obtain a reward, may have been like social loafing, in which people exert less effort to achieve the goal when they work in groups than when they work alone (Karau and Williams, 1993). These results indicate that an egalitarian outcome (rewarded or unrewarded) in competition reduces monkeys' motivation.

The analysis of monkey gaze shifts indicated that each monkey paid attention to the rival when the monkey did not receive a reward (Figure 3). Considering behavioral studies indicating that non-human primates care about what the other subject obtained (Brosnan and De Waal, 2003; Brosnan et al., 2005; Chang et al., 2011; Azzi et al., 2012), monkeys in the present study appeared to be interested in whether or not the opponent obtained a reward.

As behavioral performance differed between the normal and egalitarian reward contingencies in competition, so did activities of LPFC neurons. Black (W+L−) type and Win/Lose x Black type neurons showed activities reflecting the normal competitive reward condition. Previous studies found that LPFC neurons show context-dependent activities (White and Wise, 1999; Wallis et al., 2001; Watanabe et al., 2002; Amemori and Sawaguchi, 2006a; Kennerley and Wallis, 2009b), which are related to coding cognitive and/or motivational context information. Since Black type neurons did not distinguish the presence or absence of a reward, they may be concerned with monitoring the current normal competitive reward contingency. Win/Lose × Black type neurons, on the other hand, may be concerned with integrating the reward information (the presence or absence of a reward) with the current normal competitive reward contingency. Another possibility for Win/Lose × Black type neurons is that they may reflect the utility (subjective value) of the reward. Our behavioral data (Figure 2) indicates that the reward value in W+L− trials, where the monkey was highly motivated to win the competition, might be higher than that in W+L+ trials. LPFC neurons are known to show differential activity depending on whether or not a monkey expects and/or obtains a more preferable reward (Watanabe, 1996; Leon and Shadlen, 1999; Hikosaka and Watanabe, 2000; Amemori and Sawaguchi, 2006a; Ichihara-Takeda and Funahashi, 2008; Kim et al., 2008), and depending on whether or not a monkey anticipates the absence of a more preferable reward (Watanabe et al., 2002). Thus, the activity of Win/Lose x Black type neurons may reflect the heightened value of the reward in W+L− trials. Different from egalitarian reward (Green and Blue) trials where the monkey surely could or could not obtain a reward, the monkey was unsure whether it would receive a reward in Black trials. Thus, there is also a possibility that Win/Lose × Black neurons reflect uncertainty in obtaining the reward, or reward prediction error.

Green (W+L+) and Blue (W−L−) type neurons showed activities reflecting the egalitarian reward conditions. As Black (W+L−) type neurons may be concerned with monitoring the current normal competitive reward contingency, Green (W+L+) and Blue (W−L−) type neurons may be involved in monitoring the current egalitarian reward contingency regarding whether both the winner and loser (W+L+ trials) are given a reward, or neither of them is (W−L− trials) (Figures 4D,E). In egalitarian-outcome trials, the monkeys may prefer to wait for the competitor to win the game and then terminate the current unpreferred trial. Even in these trials, however, the monkeys overcame the tendency to simply wait for the competitor to win and tried to shoot the target, although they responded with slower and less accurate performance. Indeed, in W+L+ trials it was more advantageous for the monkey to obtain the reward much earlier by winning rather than by waiting for the competitor to win. In W−L− trials, if neither monkey won the game, the next trial was the same W−L− trial. To terminate the current W−L− trial and to advance to the next trial as quickly as possible, it was also more advantageous for the monkey to win the game than simply wait for the competitor to win. Thus, in our study it was better for the monkeys to cope with the inconvenient situation by overcoming the tendency to simply wait for the competitor to win. Green and Blue type neurons may be concerned with facilitating the monkey's behavior to cope with these inconvenient conditions by monitoring the current egalitarian outcome contingency.

Reward type neurons responded when the monkey received a reward (positive subtype) or when there was no reward (negative subtype) irrespective of whether the reward contingency was normal or egalitarian. However, they may be concerned not only with the presence or absence of the reward but also with whether the current situation was competitive or not, because many of them showed differential activity between the competitive and noncompetitive games (Figure 5). Thus, they may be concerned with integrating two types (reward/no-reward and competition/noncompetition) of information.

Since there are prefrontal neurons that code the correctness of one's own response (Watanabe, 1989), we expected to find neurons that exclusively code the performance (win-loss) outcome (i.e., neurons with significant for Win/Lose factor only). Contrary to our expectation, the percentage of such kind of neurons was not significantly higher than that from randomized data. Thus, LPFC neurons that were concerned with coding the performance outcome (such as Win/Lose × Black type neurons) appear to be also involved in processing the information regarding the presence or absence of the reward. In the study of Watanabe (1989), LPFC neurons that coded the correctness of one's own response were tested in the condition where the reward was not given at the time of the correct response, but was given 1.5 s after the response. Thus, these correctness-coding LPFC neurons may reflect also the expected reward that would be given later. It is speculated that LPFC neurons that appear to be exclusively concerned with cognitive operations may also play some important roles in processing reward information whenever monkeys work for reward. In our study, most of LPFC neurons were concerned with the current context: whether the current reward contingency was normal or egalitarian (Win/Lose × Black, Black, Green, and Blue type neurons) or whether the reward was given or not (Win/Lose × Black and Reward type neurons). Thus, LPFC neurons appear to code the performance (win-loss) outcome in conjunction with the current context of reward contingency. The result is consistent with the previous studies indicating that the LPFC is concerned with both cognitive (such as rule) and motivational context (White and Wise, 1999; Wallis et al., 2001; Watanabe et al., 2002; Amemori and Sawaguchi, 2006a,b; Ichihara-Takeda and Funahashi, 2008; Kennerley and Wallis, 2009b).

What should be done in future studies is to study, using the same paradigm, neuronal activity in other brain regions such as the medial prefrontal cortex, which is implicated in social functions (Decety et al., 2004; Zink et al., 2008; Marsh et al., 2009; Zahn et al., 2009; Tricomi et al., 2010). It is reported that monkeys pay attention to other individuals' actions and that a group of medial frontal neurons selectively encode others' action (Yoshida et al., 2011). And, in a socially interactive situation, monkey striatum neurons are reported to show reward-related differential activity depending on whether the reward was caused by the individual's own action or a partner's action (Baez-Mendoza et al., 2013). Eye position data (Figure 3) of the present study suggest that, the monkeys may have been interested in whether the reward delivery was caused by its own hitting response or by the competitor's in Green (W+L+) trials. Likewise, they may have been interested in whether or not the absence of a reward was caused by the competitor's hitting response in Blue (W−L−) trials. Since competition is inevitable in the natural environment, competing successfully is critically important for animal's survival. By comparing neuronal properties among different brain regions, or by applying transcranial magnetic stimulation or transcranial direct current stimulation to brain areas that are related to social behavior, we would be able to better understand the neural circuits that are involved in competitive behaviors.

In summary, our results indicate that egalitarian reward contingency in a competition affects a monkey's behavior and LPFC neuronal activity. These results are consistent with the evidence that the LPFC is concerned with reward (Watanabe, 1996; Kennerley and Wallis, 2009a,b; Kennerley et al., 2009), social (Zink et al., 2008; Fujii et al., 2009; Hosokawa and Watanabe, 2012) and context (White and Wise, 1999; Wallis et al., 2001; Watanabe et al., 2002; Amemori and Sawaguchi, 2006a,b; Ichihara-Takeda and Funahashi, 2008; Kennerley and Wallis, 2009b) information, and they indicate that the LPFC may play an important role in monitoring the current reward contingency and in integrating it with the performance (win-loss) outcome to better adapt to competitive situations, and thus for better survival.

### Conflict of interest statement

The authors declare that the research was conducted in the absence of any commercial or financial relationships that could be construed as a potential conflict of interest.
